# Production of the versatile cellulase for cellulose bioconversion and cellulase inducer synthesis by genetic improvement of *Trichoderma reesei*

**DOI:** 10.1186/s13068-017-0963-1

**Published:** 2017-11-15

**Authors:** Jia Gao, Yuanchao Qian, Yifan Wang, Yinbo Qu, Yaohua Zhong

**Affiliations:** State Key Laboratory of Microbial Technology, School of Life Sciences, Shandong University, Jinan, 250100 People’s Republic of China

**Keywords:** *Trichoderma reesei*, Cellulase, *cre1*, β-Glucosidase, Transglycosylation, β-Disaccharides

## Abstract

**Background:**

The enzymes for efficient hydrolysis of lignocellulosic biomass are a major factor in the development of an economically feasible cellulose bioconversion process. Up to now, low hydrolysis efficiency and high production cost of cellulases remain the significant hurdles in this process. The aim of the present study was to develop a versatile cellulase system with the enhanced hydrolytic efficiency and the ability to synthesize powerful inducers by genetically engineering *Trichoderma reesei*.

**Results:**

In our study, we employed a systematic genetic strategy to construct the carbon catabolite-derepressed strain *T. reesei* SCB18 to produce the cellulase complex that exhibited a strong cellulolytic capacity for biomass saccharification and an extraordinary high β-glucosidase (BGL) activity for cellulase-inducing disaccharides synthesis. We first identified the hypercellulolytic and uracil auxotrophic strain *T. reesei* SP4 as carbon catabolite repressed, and then deleted the carbon catabolite repressor gene *cre1* in the genome. We found that the deletion of *cre1* with the selectable marker *pyrG* led to a 72.6% increase in total cellulase activity, but a slight reduction in saccharification efficiency. To facilitate the following genetic modification, the marker *pyrG* was successfully removed by homologous recombination based on resistance to 5-FOA. Furthermore, the *Aspergillus niger* BGLA-encoding gene *bglA* was overexpressed, and the generated strain *T. reesei* SCB18 exhibited a 29.8% increase in total cellulase activity and a 51.3-fold enhancement in BGL activity (up to 103.9 IU/mL). We observed that the cellulase system of SCB18 showed significantly higher saccharification efficiency toward differently pretreated corncob residues than the control strains SDC11 and SP4. Moreover, the crude enzyme preparation from SCB18 with high BGL activity possessed strong transglycosylation ability to synthesize β-disaccharides from glucose. The transglycosylation product was finally utilized as the inducer for cellulase production, which provided a 63.0% increase in total cellulase activity compared to the frequently used soluble inducer, lactose.

**Conclusions:**

In summary, we constructed a versatile cellulase system in *T. reesei* for efficient biomass saccharification and powerful cellulase inducer synthesis by combinational genetic manipulation of three distinct types of genes to achieve the customized cellulase production, thus providing a viable strategy for further strain improvement to reduce the cost of biomass-based biofuel production.

**Electronic supplementary material:**

The online version of this article (10.1186/s13068-017-0963-1) contains supplementary material, which is available to authorized users.

## Background

Lignocellulosic biomass, especially from crop and forest residues, is among the most abundant, but under-utilized resources on Earth [1, 2]. It is now gaining wide attention as a sustainable source for production of environment-friendly biofuels such as cellulosic ethanol [3, 4]. Cellulolytic enzymes produced by microorganisms play a key role in the bioconversion process, but the cost of enzymes remains one of the major hurdles to the development of an economically viable cellulosic ethanol on an industrial scale [5]. The filamentous fungus *Trichoderma reesei* has the ability to secrete large amounts of cellulolytic enzymes, and thus is the main industrial source for cellulase production. The cellulolytic system of *T. reesei*, which can efficiently degrade insoluble cellulose into glucose, comprises three classes of enzymes: cellobiohydrolases (CBHs), endoglucanases (EGs), and β-glucosidases (BGLs) [6]. In addition, lytic polysaccharide monooxygenases (LPMOs) are a recently discovered class of enzymes capable of oxidizing recalcitrant cellulose substrates and boosting the activity of classical cellulolytic enzymes [7, 8]. However, these enzymes are conditionally expressed, and one of the master regulators is the Cys-2/His-2 (C2H2) zinc finger transcription factor Cre1, which mediates the carbon catabolite repression (CCR) [9]. It downregulates the expression of enzymes involved in the catabolism of carbon sources other than the preferred ones such as glucose, and most of these enzymes are belonging to the cellulolytic enzymes [10]. In fact, the hypercellulolytic mutant RUT-C30 derived from the wild-type strain QM6a has a truncated version of Cre1, which is one of the main causes for the improved cellulase production [11]. Further deletion of the *cre1* gene could significantly elevate expression of the cellulolytic enzymes under both inducing and noninducing conditions [12]. Therefore, Cre1 represents a valid engineering target to improve cellulase production in *T. reesei* [10].

In the *T. reesei* cellulolytic system, BGL could hydrolyze cellobiose to glucose in the final step of cellulose degradation and relieve the feedback inhibition of cellobiose on CBHs and EGs [13, 14]. It is generally recognized that the insufficiency of BGL in this cellulase complex is one of the bottlenecks in efficient cellulose hydrolysis [15, 16]. Therefore, a major challenge in the biomass conversion is obtaining the optimal amount of BGL to complete the cellulose hydrolysis [17, 18]. Supplementation of the *T. reesei* cellulase preparation with external BGL has been shown to improve glucose production from cellulose degradation, but additional complexity is also introduced due to the in vitro preparation of BGL [19, 20]. Recently, construction of engineered strains by overexpressing homologous and heterogenous *bgl* genes has been the preferred strategy to address this problem [21–23]. The endogenous *bgl1* gene under the control of different strong promoters was transformed into *T. reesei*, and the transformants displayed BGL activities ranging from 0.48 to 8.30 IU/mL [15, 18, 23]. Particularly, the BGL enzymes from several *Aspergillus* and *Penicillium* species were reported to exhibit relatively higher specific activities, and studies have shown that heterogenous expression of these genes in *T. reesei* could significantly increase BGL activity (up to 34.31 IU/mL), resulting in the enhanced efficiency of cellulose hydrolysis [20, 24].

In addition, majority of cellulases in *T. reesei* are adaptive enzymes, and their full expression requires the presence of an inducer. Although cellulose can be used as the cellulase-inducing carbon source, cellulose itself is practically unable to trigger the induction because of its insolubility [25]. Currently, one acceptable model for cellulase induction is that the extracellular cellulose is hydrolyzed by the constitutive cellulases to release small amounts of cello-oligosaccharides such as cellobiose, acting as inducers for the further large-scale synthesis of cellulases [26]. However, high cost of pure celluloses such as Avicel and low mycelial growth with cellulose as carbon source make cellulase production cost very high [27, 28]. Soluble β-disaccharides, including sophorose and lactose, are found to be not only the suitable carbon sources but powerful inducers for cellulase production in *T. reesei* [29–31]. Nevertheless, sophorose rarely exists in nature, and is extremely expensive to manufacture and purify, thus limiting its industrial application for cellulase production [30, 31]. Lactose is easily available but less effective in cellulase induction than sophorose [28, 30, 32]. A growing number of studies have shown that BGLs possess the transglycosylation property, besides the hydrolytic activity, to synthesize sophorose [25, 33, 34]. For example, the purified β-glucosidase Td2F2 and the commercial β-glucosidase from *A. niger* (NS50010) were reported to catalyze transglycosylation to produce sophorose, cellobiose, and gentiobiose at high concentration of glucose [28, 35]. Specifically, Li et al. confirmed that the cellulase activity of *T. reesei* RUT-C30 could be remarkably enhanced by using the transglycosylation product as inducer [28]. However, this type of inducer is obtained from purified or commercial enzymes, which would increase the production cost [36]. Therefore, construction of the *T. reesei*-engineered strains with high β-glucosidase activity would contribute to synthesizing the soluble cellulase-inducing oligosaccharides for low-cost production of cellulases.

In our previous study, we identified a hypercellulolytic variant *T. reesei* SN1 showing relatively high EG activity but low BGL activity, and constructed a uracil auxotrophic strain SP4 from SN1 through genetic engineering for strain improvement [18]. In this study, multiple gene manipulations, including deletion of *cre1*, elimination of the marker gene *pyrG,* and overexpression of the *A. niger* BGLA-encoding gene *bglA* in *T. reesei* SP4, were adopted. The cellulase complex produced by the engineered strain SCB18 exhibited remarkably high BGL activity (103.9 IU/mL), which was 51.3-fold higher than that of its parental strain. Subsequently, it was not only used for efficient saccharification of cellulosic substrates, but also applied to synthesize β-disaccharides directly from glucose through the transglycosylation reaction, which were further utilized as inducers for cellulase production.

## Results and discussion

### Deletion of the carbon catabolite repressor gene *cre1* in *T. reesei* SP4

**Fig. 1:**
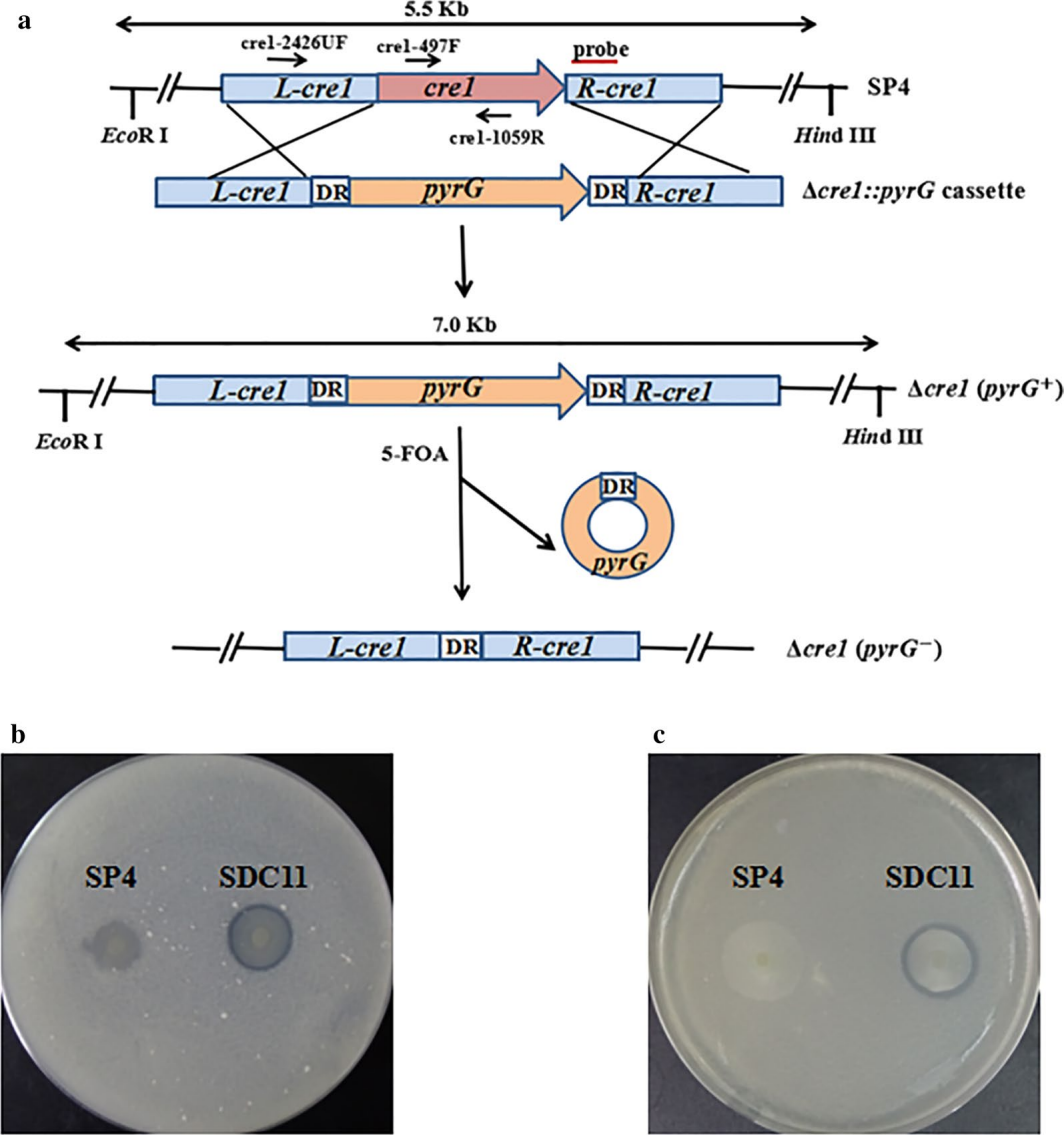
Deletion of the *cre1* gene in *T. reesei* SP4. **a** Deletion of *cre1* and excision of the *pyrG* marker in the Δ*cre1* strains. **b** Growth of *T. reesei* SP4 and Δ*cre1* strain SDC11 on the MM plate containing Avicel (0.5%) as sole carbon source. **c** Growth of *T. reesei* SP4 and SDC11 on the MM plate containing both glucose (1.0%) and Avicel (0.5%) as carbon sources

Cre1 and its homologs are known to be the master regulators of carbon assimilation in filamentous fungi, allowing the cell to prefer the assimilation of carbon sources of high nutritional value over others [9, 37]. In the cellulolytic fungi, Cre1 has been shown to be a key repressor involved in cellulase gene expression [12, 38, 39]. Based on previous studies, most of the hypercellulolytic mutant strains are carbon catabolite derepressed, such as *T. reesei* RUT-C30 and PC-3-7 [11, 40]. In this study, the hypercellulolytic *T. reesei* strain SP4 was found to be carbon catabolite repressed and contained the full-length Cre1-encoding gene *cre1* (Additional file 1: Figure S1). To relieve the genes encoding the cellulolytic enzymes from CCR in *T. reesei* SP4, the Δ*cre1::pyrG* cassette was constructed and transformed into SP4 to delete *cre1* via replacing it with the *A. niger pyrG* marker gene (Fig. 1a). The generated Δ*cre1* (*pyrG* ^+^) strain SCP11 was analyzed through amplification of the full-length and internal fragments of the *cre1* gene (Additional file 2: Figure S2a), and the absence of *cre1* was further confirmed by its growth phenotype on the media containing 0.5% Avicel and 1.0% glucose (data not shown). The *Eco*R I/*Hin*d III-digested genomic DNA was hybridized with the *cre1* probe for further Southern blot assay, which yielded a 7.0-kb fragment for the strain SCP11 and a 5.5-kb fragment for the parental strain SP4 (Additional file 2: Figure S2b). These results suggested that the *cre1* gene was successfully knocked out in SCP11. To facilitate further strain improvement via genetic manipulation, the marker gene *pyrG* was required to be removed, which could be done by homologous recombination between two direct repeats in the Δ*cre1::pyrG* cassette. Here, SCP11 was grown on resistance to 5-FOA to excise the *pyrG* marker gene (Fig. 1a). Finally, the Δ*cre1* (*pyrG* ^−^) strain SDC11 was achieved, which was found to have lost the *pyrG* gene in the genome via PCR analysis with the prime pair, pyrG-F/pyrG-R (Additional file 1: Figure S1a). To confirm that SDC11 is a uracil auxotrophic strain, SDC11 was grown on minimal media (MM) plates with or without uracil (Additional file 1: Figure S1b, c). It was found that SDC11 and the control strain SP4 could grow well on MM plates with uracil, but no colonies were formed without uracil, while its parental strain SCP11 could grow well on MM plates with or without uracil. These results indicated that the *pyrG* marker gene was successfully excised in the SDC11 genome, and the SDC11 strain regained the uracil auxotrophy. To further test whether *T. reesei* SDC11 is also carbon catabolite derepressed, it was grown on the MM plates containing Avicel and glucose or only Avicel as the carbon sources (Fig. 1b, c). In both plates, clear cellulolytic halos were observed around the colonies of SDC11, but the control strain SP4 could not exhibit any cellulolytic halo, indicating that SDC11 was carbon catabolite derepressed. In addition, deletion of *cre1* led to relatively lower radial growth rate, fewer aerial hyphae, and less production of spores in *T. reesei* SDC11 compared with SP4 (data not shown). It was reported that the absence of *cre1* could significantly affect the colony morphology of *T. reesei* QM6a whether in the glucose-containing medium causing catabolite repression or in the medium inducing cellulase expression [12]. Besides, decreased growth rate and reduced sporulation were also exhibited in the *A. niger* Δ*creA* mutant, where the CreA-encoding gene *creA*, the homolog of *T. reesei cre1*, was disrupted [41]. Collectively, these results illustrated that the carbon catabolite repressor gene *cre1* in *T. reesei* was successfully deleted in the strain SDC11.

### Cellulase production and its cellulolytic potential in biomass saccharification by *T. reesei* SDC11

**Fig. 2:**
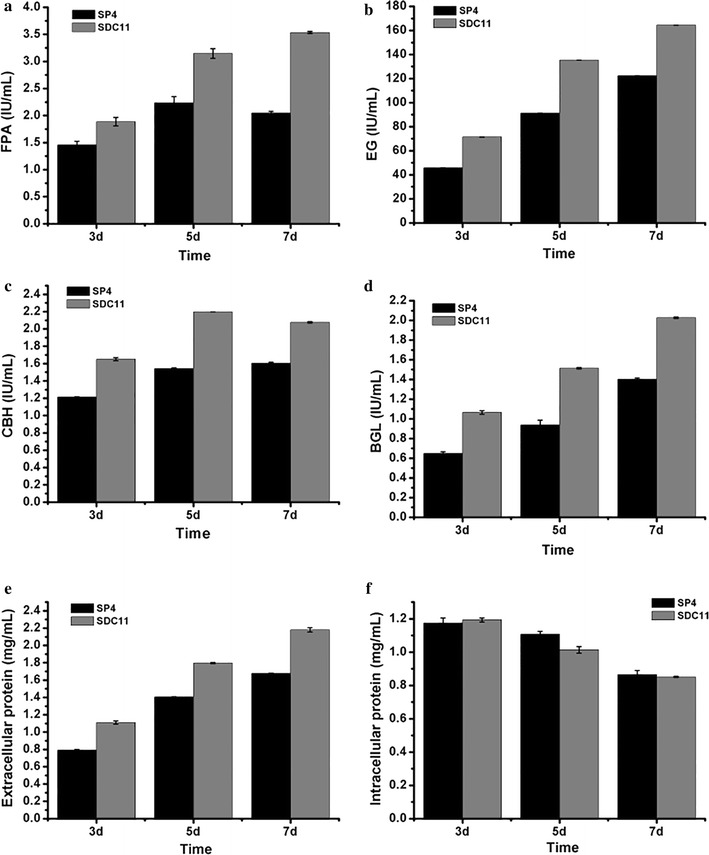
Cellulase production by the Δ*cre1* strain SDC11. FPA activity (**a**), EG activity (**b**), CBH activity (**c**), BGL activity (**d**), extracellular protein (**e**) and intracellular protein (**f**) were measured at 3d, 5d, and 7d of cellulase-inducing cultivation. Data are the means of three independent experiments; error bars show standard deviations

**Fig. 3:**
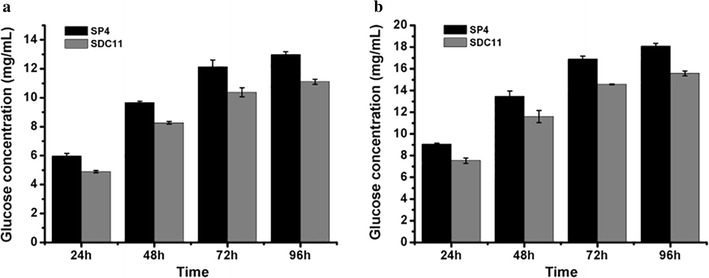
Saccharification of differently pretreated concob residues by *T. reesei* SDC11. **a** Glucose released from saccharification of acid-pretreated corncob residue every 24 h. **b** Glucose released from saccharification of delignified corncob residue every 24 h. Data are the means of three independent experiments; error bars show standard deviations

To investigate the capacity to secrete cellulase, SDC11 and the control strain SP4 were fermented in CPM for 7d, and the fermentation broths were used for cellulase activity assay. As shown in Fig. 2a, *T. reesei* SDC11 exhibited 72.6% higher total cellulase activity (FPA) than that of SP4 at the end of fermentation. To verify the contribution of the absence of *cre1* to FPA increase in SDC11, the individual cellulase activities of EG, CBH, and BGL were assayed and compared to those of SP4. It was found that all of these enzymatic activities were remarkably enhanced. Specially, EG, CBH, and BGL activities showed 48.3, 42.4, and 44.6% increases after 7d of cellulase-inducing cultivation, respectively (Fig. 2b–d). While the amount of total secreted protein was detected, SDC11 produced no more than 30.0% increase compared with SP4 (Fig. 2e). In addition, the growth rate of SDC11, which was measured by detecting the total intracellular protein, was slightly lower than that of SP4 (Fig. 2f). These results indicated that the specific cellulase activities in the Δ*cre1* strain SDC11 were improved, and this improvement was not related to the fungal growth rate. Then, the saccharification potential of the SDC11 enzyme for converting cellulosic materials was examined. Two types of pretreated corncob materials, the acid-pretreated corncob residue and the delignified corncob residue, were adopted for enzymatic hydrolysis at 50 °C and pH 4.8 for 96 h. The amounts of glucose released from these two materials by SDC11 and SP4 observably increased with time increasing from 24 to 96 h. However, with equal FPA loading, the released glucose by the SDC11 enzyme was lower than that for SP4. Specifically, in the saccharification of acid-pretreated corncob residue, the released glucose by SDC11 at the end of saccharification was 11.1 mg/mL (corresponding to 31.8% cellulose conversion), which was lower than the value for SP4 (13.0 mg/mL, corresponding to 37.3% cellulose conversion) (Fig. 3a). When the delignified corncob residue was used as substrate, the final glucose amount (15.6 mg/mL, corresponding to 44.7% cellulose conversion) yielded by SDC11 after a 96-h reaction was also lower than that by SP4 (18.1 mg/mL, corresponding to 51.9% cellulose conversion) (Fig. 3b). Mooney et al. reported that the presence of lignin could act as a steric hindrance for cellulose accessibility to cellulolytic enzymes [42]. The acid-pretreated corncob residue used here still contained large quantities of lignin components (17.7%), and another substrate, the delignified corncob residue, contained little lignin (3.2%) after pretreatment [43]. Thus, the amount of released glucose via enzymatic hydrolysis of the acid-pretreated material was significantly lower than that of the delignified one under the equal cellulase loading condition. Remarkably, although *T. reesei* SDC11 exhibited dramatically enhanced FPA activity, its saccharification efficiency for cellulosic materials was slightly lower than that of SP4. The main reason for the discrepancy between the cellulase activity and the saccharification efficiency was probably due to the relatively reduced amount of extracellular BGL in the cellulase complex, as the ratio of BGL activity to FPA in SDC11 was 0.57, which was lower than that in SP4 (0.68). The lower BGL ratio in SDC11 was further confirmed by renaturing SDS-PAGE analysis with equal FPA loading (Additional file 3: Figure S3).

In filamentous fungi *T. reesei* and *N. crassa*, Cre1/CRE-1 functions as a global transcription factor and affects gene expression directly or indirectly [44, 45]. The functions of enzymes encoded by the Cre1/CRE-1-regulated genes include lignocellulosic biomass-degrading, nitrogen-uptaking, developmental processes, chromatin remodeling, and so on [46]. In this study, the CBH activity of the control strain SP4 was remarkably reduced compared to that of SDC11 (Fig. 2c). The reason might be that Cre1 could bind to two closely spaced 5′-CCCCAC-3′ motifs in the *cbh1* promoter region and then directly represses *cbh1* transcription in the *cre1* ^+^ strain but not in the Δ*cre1* strain [46, 47]. Moreover, *cbh2* is found to be not Cre1 regulation dependent as some evidences indicate that CCR is only conferred by double Cre1-binding sites and only a single putative Cre1-binding site exists in the *cbh2* promoter region in *T. reesei* [39, 48–50]. However, Cre1 could indirectly suppress *cbh2* expression via regulating Xyr1, the major activator of the cellulase-encoding genes [50]. Thus, the CBH activity in SDC11 could be speculated to be enhanced due to the absence of Cre1. The transcriptional regulation of *egl1* is shown to be strictly dependent on Xyr1 and repressed by Cre1 indirectly, like *cbh2* [39, 49]. Therefore, deletion of *cre1* also caused the significant enhancement of EG activity in SDC11 (Fig. 2b). Besides, studies have indicated that *bgl1* encoding the extracellular β-glucosidase BGL1 is also suppressed by Cre1 indirectly [48, 49, 51]. Furthermore, the absence of *cre1* in the hypercellulolytic strain QM9414 resulted in a significant increase in the amount of BGL [51]. In this study, the BGL activity in the Δ*cre1* strain SDC11 constructed was also improved (Fig. 2d), which was in accordance with the above-mentioned reports. In our previous study, BGL activity was positively correlated with the cellulose conversion and had a dramatic influence on the enzymatic hydrolysis of corncob residues [15, 18]. Therefore, the relatively reduced ratio of BGL activity to FPA in the cellulase complex produced by SDC11 caused the decreased saccharification efficiency toward the cellulosic materials (Fig. 3).

### Construction of the *A. niger* BGLA-overexpressing strain SCB18 and characterization of its cellulase production

**Fig. 4:**
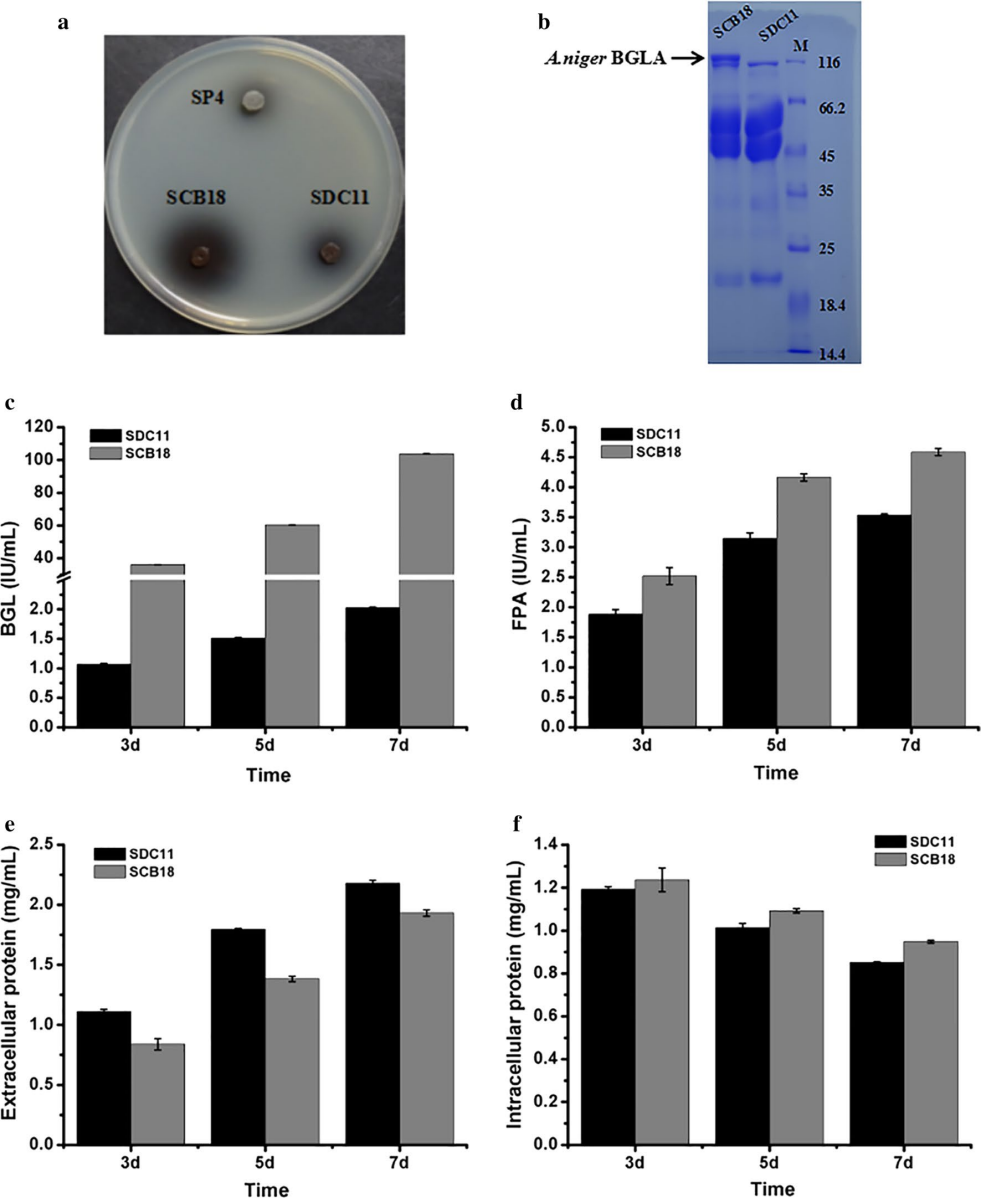
Overexpression of the *A. niger* BGLA-encoding gene *bglA* in *T. reesei* SDC11. **a** Detection of β-glucosidase activity on the CMC-esculin plate. **b** SDS-PAGE analysis of the fermentation broth during cellulase-inducing cultivation. **c**, **d** The BGL and FPA activities produced during cellulase-inducing cultivation, respectively. **e**, **f** The extracellular protein and intracellular protein that were measured at 3d, 5d, and 7d of cellulase-inducing cultivation, respectively. Data are the means of three independent experiments; error bars show standard deviations

Although *T. reesei* is one of the best fungal cellulase producers, the amount of BGL secreted by this fungus is insufficient for effective hydrolysis of cellobiose, thus resulting in the inhibition of the production in the *T. reesei* enzyme system [52]. Due to this restriction on saccharification imposed by deficiency of BGL, the efficiency of enzymatic hydrolysis cannot be improved much by increasing enzyme loading. In general, enzymatic saccharification of biomass with considerable efficiency can be acquired by supplementation of the commercial *A. niger* BGL, such as Novozym 188 preparation [19]. Sukumaran et al. evaluated the applications of *T. reesei* cellulase and *A. niger* BGL for biomass hydrolysis and demonstrated the feasibility of this combination for efficient hydrolysis of at least three biomass residues [53]. It is found that *A. niger* BGL has a tadpole-like structure consisting of catalytic domain (CD) and fibronectin III-like domain (FnIII) connected by a long linker and exhibits less adsorption onto lignin than *T. reesei* BGL, which indirectly facilitates enzymatic hydrolysis of cellulose due to increased hydrolysis of cellobiose that in turn accumulates and inhibits CBHs/EGs [54, 55]. Here, to overcome the lack of BGL in the SDC11 enzyme system, an overexpressing cassette containing the *A. niger bglA* gene under the control of the *T. reesei cbh1* promoter was constructed and co-transformed with the *pyrG*+DR fragment into *T. reesei* SDC11 (Additional file 4: Figure S4a). The ability of the candidate transformants to secrete BGL was first detected by screening on CMC-esculin plates (data not shown). And one transformant, SCB18, which showed much larger black zone around the colony than its parental strain SDC11, was selected (Fig. 4a). Then it was verified through PCR amplification of the chimeric fragment spanning the *cbh1* promoter and the *bglA* gene with primer pair cbh1-1138UF/bg-R as well as the internal fragment of the *bglA* gene with primer pair bg-F/bg-R (Additional file 4: Figure S4b). To investigate the capacity to secrete cellulase, SCB18 was grown on the MM plates containing Avicel and glucose or only Avicel as the carbon sources (Additional file 4: Figure S4c, d). In both the plates, clear cellulolytic halos were observed around the colony of SCB18 and its parental strain SDC11, but the control strain SP4 (glucose repression-sensitive) could not exhibit any cellulolytic halo. To further analyze its cellulase production ability, SCB18 was fermented in CPM for 7d, and the fermentation broth was used for cellulase activity assay. First, SDS-PAGE analysis was applied to detect the secreted proteins from the fungal strains. A clear band of approximately 120 kDa (the expected molecular weight of *A. niger* BGLA), which was not present in SDC11, was observed in the secreted proteins of SCB18 (Fig. 4b). This result indicated that the *A. niger bglA* gene was successfully expressed in SCB18. In accordance with the above result, the BGL activity of SCB18 was significantly improved and reached up to 103.9 IU/mL at the end of fermentation, which was 51.3-fold higher than that of its parental strain SDC11 (Fig. 4c). In this study, *A. niger* BGLA was overexpressed under the control of *T. reesei cbh1* promoter that is a strong promoter for endogenous/heterogeneous proteins production [56]. In addition, it was known that *A. niger* BGL had much higher specific activity (198.5 IU/mg) toward pNPG than that of *T. reesei* (88.5 IU/mg) [57, 58]. These may be the reasons behind the improvement of BGL activity in SCB18. In our previous study, the native *bgl1* gene was overexpressed using a modified *cbh1* promoter in *T. reesei* SP4, and a BGL activity of 8.3 IU/mL was obtained in the recombinant strain [18]. Ma et al. reported that the *P. decumbens bgl1* gene was successfully expressed under the control of the *cbh1* promoter in *T. reesei* RUT-C30, and the recombinant strain exhibited a BGL activity of 34.3 IU/mL [20]. To our knowledge, the BGL activity (103.9 IU/mL) produced by SCB18 is the highest BGL activity reported for *T. reesei*. Furthermore, *T. reesei* SCB18 provided a 29.8% higher FPA activity (4.6 IU/mL) than that of the parental strain SDC11 (3.5 IU/mL) (Fig. 4d). This result is consistent with the recent reports that high BGL activity could promote hydrolysis of the filter paper substrate [59–61]. The comparison of BGL activity and cellulase titer achieved in this study to those produced by other *T. reesei* BGL-overexpressing strains is highlighted in Additional file 5: Table S1. While the amount of total secreted protein was detected, SCB18 produced 11.4% decrease compared with SDC11 (Fig. 4e). In addition, the growth rate of SCB18, which was measured by detecting the total intracellular protein, was slightly higher than SDC11 (Fig. 4f). Consequently, the cellulase preparation of SCB18 exhibited extremely high specific BGL activity and also possessed demonstrable cellulolytic ability.

### Saccharification of corncob substrates by the cellulase preparation from *T. reesei* SCB18

**Fig. 5:**
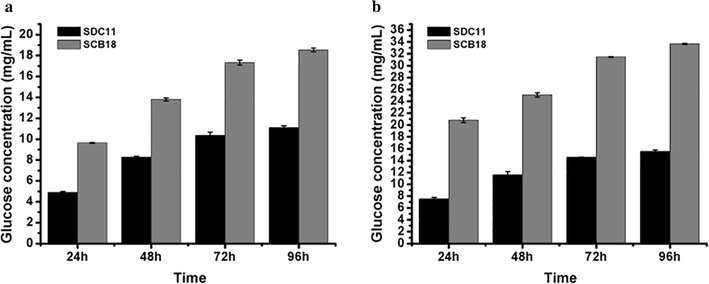
Saccharification of different pretreated concob residues by the BGLA-overexpressing strain SCB18. **a** Glucose released from saccharification of acid-pretreated corncob residue every 24 h. **b** Glucose released from saccharification of delignified corncob residue every 24 h. Data are the means of three independent experiments; error bars show standard deviations

The cellulase preparation produced by *T. reesei* SCB18 was utilized to investigate the influence of overexpression of *A. niger* BGLA on hydrolyzing the pretreated corncob residues. In the saccharification of acid-pretreated corncob residue, the released glucose by SCB18 (18.5 mg/mL, corresponding to 53.1% cellulose conversion) was significantly higher than the value for SDC11 (11.1 mg/mL, corresponding to 31.8% cellulose conversion) after a total enzymatic reaction of 96 h (Fig. 5a). When the delignified corncob residue was used as substrate, the final glucose released by SCB18 after a 96-h reaction was 33.7 mg/mL (corresponding to 96.6% cellulose conversion), which was also remarkably higher than that by SDC11 (15.6 mg/mL corresponding to 44.7% cellulose conversion) (Fig. 5b). On the other hand, Chen et al. reported that when the ratio of BGL activity to FPA activity was improved to 2 by adding *P. decumbens* BGL1 or *A. niger* BGLA, the saccharification ability toward cellulosic materials could be improved [62]. In our previous study, the ratio of BGL to FPA was enhanced to 2.8 by overexpression of the native BGL1, and the saccharification capability toward corncob residues was significantly improved [18]. In this study, the ratio of BGL to FPA in SCB18 reached 22.6, which was much higher than that in SDC11 (0.57), and efficient enzymatic hydrolysis of corncob substrates was successfully achieved. Taken together, the cellulase preparation produced by *T. reesei* SCB18, which was obtained through a two-step gene-manipulation process containing deletion of *cre1* and overexpression of *A. niger* BGLA, exhibited superior performance on saccharification of differently pretreated corncob substrates, indicating its potential applications in cellulose bioconversion.

### β-Disaccharides synthesis by the crude enzyme preparation from *T. reesei* SCB18 using glucose as substrate

**Fig. 6:**
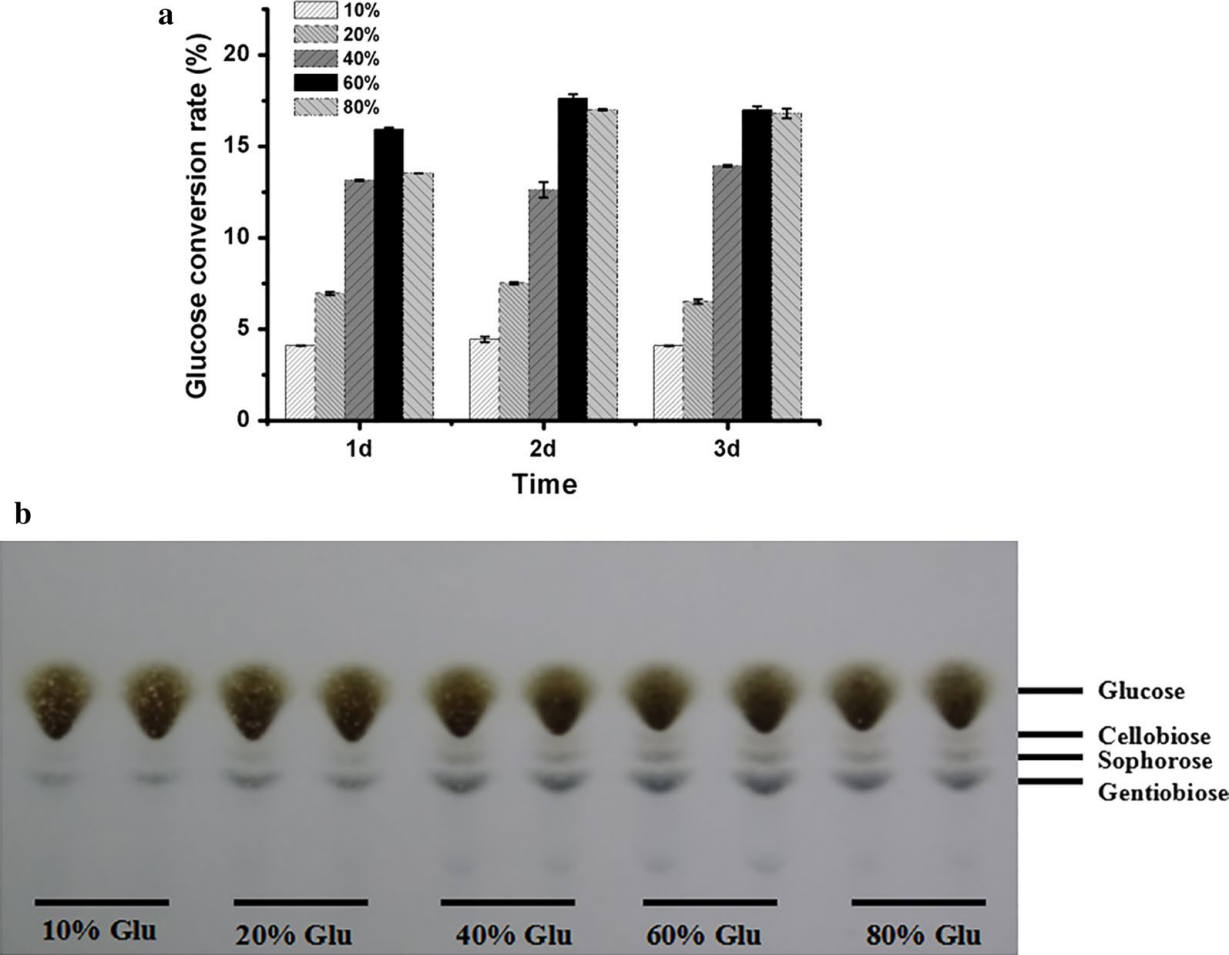
Transglycosylation capability of the fermentation broth produced by *T. reesei* SCB18 under different glucose concentrations. **a** Glucose conversion rate (%) by determining glucose residual content at 1d, 2d, and 3d of transglycosylation reaction. **b** Analysis of the transglycosylation products containing glucose and β-disaccharides by thin-layer chromatography (TLC) at 2d. Data are the means of three independent experiments; error bars show standard deviations

Many researches have proven that BGLs from different sources, in addition to possessing the property of hydrolyzing cellobiose, could catalyze the formation of more complex β-oligosaccharides in vitro through transglycosylation reaction using a wide range of substrates such as glucose, cellobiose, and gentiobiose [63–65]. However, these BGLs present transglycosylation activity only when the concentration of the substrates is relatively high [66–68]. In order to investigate the transglycosylation capacity of the fermentation broth produced by *T. reesei* SCB18, which held an extraordinary BGL activity (103 IU/mL), the transglycosylation assay was performed with different concentrations of glucose [10, 20, 40, 60, and 80% (w/v)] as substrates. The transglycosylation reaction was conducted at 65 °C and pH 4.8 for 3d. As shown in Fig. 6a, the glucose conversion rate was significantly improved with the concentration of glucose increasing from 10 to 60%, while the conversion rate of 80% glucose was lower than that of 60% glucose. Notably, the glucose conversion rate reached the highest value (17.6%) when transglycosylation reaction was performed with 60% glucose for 2d. Then, TLC assay was applied to further analyze the transglycosylation product after 2-d reaction. As shown in Fig. 6b, two β-disaccharides, sophorose and gentiobiose, were the main products regardless of the concentration of glucose, and their amounts increased with the increased concentration of glucose from 10 to 60%. Cellobiose could also be observed when the concentration of glucose was more than 40%. In accordance with the glucose conversion rate, the amount of the transglycosylation product with 60% glucose as substrate was the highest among the product detected. It is known that BGLs are economically enticing for β-oligosaccharide synthesis compared with glycosyltransferases, but their transglycosylation products are altered depending on the initial substrates [63, 69]. For instance, cellotriose and gentiobiose could be detected after the incubation of *A. niger* BGL with high concentration of cellobiose. While using the same substrate, cellotetraose and cellotriose were synthesized after being catalyzed by a *Trichosporon asahii* BGL [63, 69, 70]. Gentiobiose could be synthesized by BGL from *Thermus caldophilus* GK24 via transglycosylation reaction with high concentration of glucose as substrate [71], while cellobiose, cellotriose, and cellotetraose were obtained after the incubation of a *Trichosporon asahii* BGL with high concentration of glucose [63]. More recently, cellobiose, sophorose, and gentiobiose were synthesized by commercial BGL via transglycosylation reaction with 60% glucose after 3d of cultivation [28]. In this study, cellobiose, sophorose, and gentiobiose were also detected, and their contents reached the peak values after incubation of a crude enzyme preparation with 60% glucose at 2d, indicating that the cellulase preparation with high BGL activity produced by *T. reesei* SCB18 could be used for β-disaccharides synthesis.

### The β-disaccharides synthesized by the crude enzyme preparation from *T. reesei* SCB18 as efficient inducers for cellulase production

**Fig. 7:**
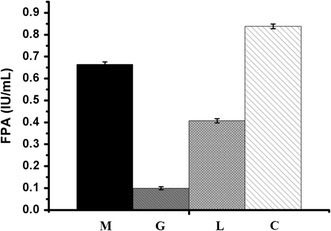
Cellulase production by *T. reesei* SDC11 using the transglycosylation product as inducer. Comparison of FPA activities using different carbon sources at the same concentration (2%) after 3d of cultivation. M, G, L, and C represent the transglycosylation product, glucose, lactose, and cellulose, respectively. Data are the means of three independent experiments; error bars show standard deviations

Various β-disaccharides, including cellobiose, sophorose, and gentibiose, have been acknowledged as powerful inducers for cellulase expression in filamentous fungi. For example, sophorose can induce 2500-fold more cellulase than cellobiose in *T. reesei*, while gentiobiose was found to induce 50 times more cellulase in *P. purpurogenum* in the presence of BGL inhibitor [25, 72]. Here, to analyze the ability of the sugar mixture synthesized by the transglycosylation reaction to induce cellulase production, the carbon catabolite-derepressed strain *T. reesei* SDC11 was fermented using different carbon sources for 3d. When the transglycosylation product was used as the inducer, the FPA activity was 63.0% higher than that with lactose, but 20.8% lower than that with cellulose (Fig. 7). That is, the transglycosylation product showed stronger cellulase-inducing effect than lactose, but lower than that with cellulose. When glucose was used as the carbon source, a small amount of FPA activity could also be measured, which was probably due to the alleviation of carbon catabolite repression caused by deletion of *cre1* gene in SDC11. It is known that the utilization of soluble carbon sources such as lactose, compared with the insoluble cellulose, could allow greater control of fermentation since fungal growth and enzyme generation did not rely on cellulose hydrolysis [27]. However, the cellulase yield with lactose as soluble carbon source is lower than that with cellulose [73]. Here, the soluble β-disaccharides in the transglycosylation product synthesized from glucose by the crude enzyme of SCB18 exhibited stronger cellulase-inducing ability than lactose, thus representing an alternative and prospective candidate as carbon source for larger production of economically attractive and competitive cellulase.

## Conclusions

**Fig. 8:**
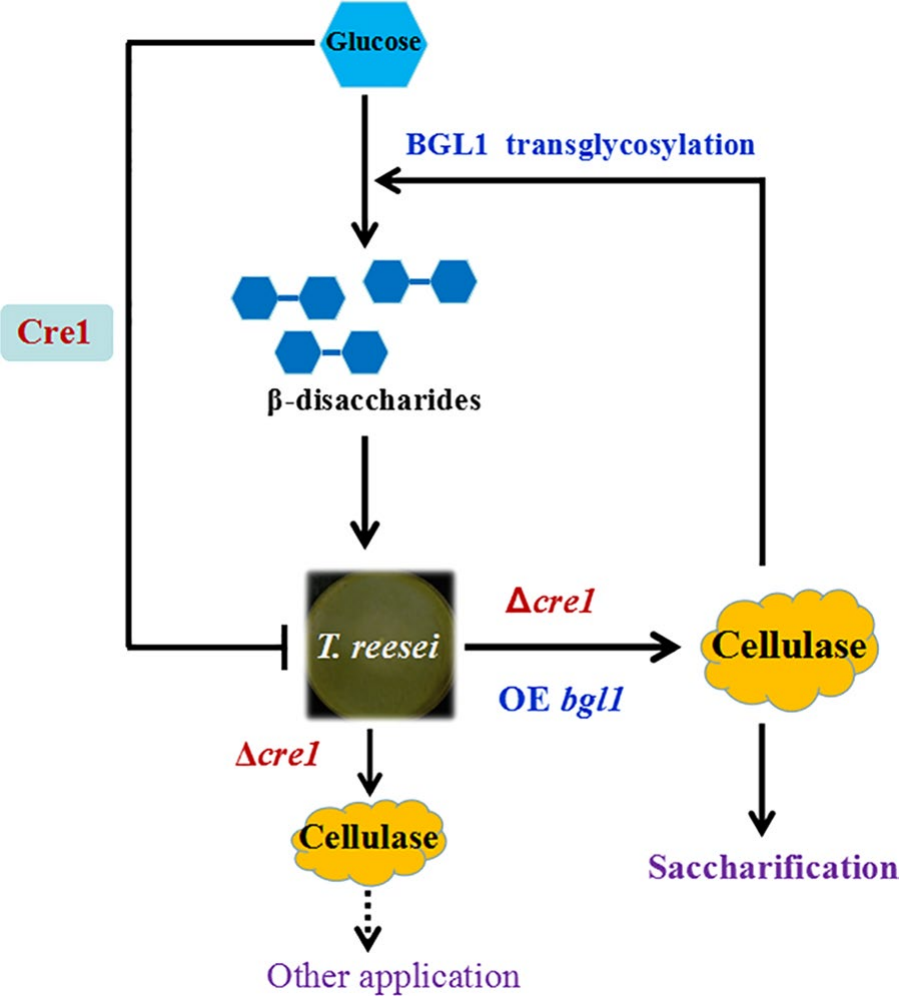
Schematic model illustrating the principle for constructing the BGLA-overexpressing cellulase system with pleiotropic functions. The cellulase expression in *T. reesei* (SP4) is repressed through the carbon catabolite repressor Cre1 when glucose is present. Deletion of the *cre1* gene results in glucose derepression and enhanced cellulase expression. Further overexpression of the *A. niger bglA* gene produces the *T. reesei* (SCB18) cellulase with high efficiency for saccharification of different cellulosic substrates. Meanwhile, the cellulase in *T. reesei* could also be induced by β-disaccharides (cellobiose and sophorose). The BGLA-overexpressing cellulase system produced by *T. reesei* (SCB18) can conversely synthesize these β-disaccharides with its transglycosylation activity from glucose as carbon source, which could be further used as cellulase inducers

In this study, we used multiple genetic manipulations to construct a versatile cellulase system for enhancing cellulose saccharification and synthesizing cellulase inducers based on a hypercellulolytic *T. reesei* strain (Fig. 8). The *cre1* gene was first deleted to relieve carbon catabolite repression in *T. reesei* SP4 and further construct an engineered strain *T. reesei* SCB18 with extremely high BGL activity. The cellulase system exhibited remarkably strong cellulolytic ability toward differently pretreated corncob residues and high capacity to synthesize β-disaccharides from glucose through transglycoslation reaction, which showed superior cellulase-inducing ability relative to lactose. Consequently, this study develops a BGL-overexpressing cellulase system with pleiotropic functions for application in cellulose bioconversion and suggests a new prospective strategy for strain improvement of industrial fungi.

## Methods

### Strains, plasmids, and culture conditions

*Trichoderma reesei* SP4, a uracil auxotrophic strain derived from the hypercellulolytic variant *T. reesei* SN1 [18], was used as the initial host for strain improvement. The well-known glucose repression-sensitive strain *T. reesei* QM9414 (ATCC 26921) and another cellulase high-producing strain RUT-C30 (ATCC 56765) that is less sensitive to glucose repression were selected as the control strains. The plasmid pAB4-1 was used as the template to amplify the *Aspergillus niger pyrG* gene, a selection marker to encode orotidine-5′-phosphate decarboxylase [74]. For conidia production, all strains were grown and maintained on potato dextrose agar (PDA) plates containing peeled potato extract 200 g/L, glucose (2%, w/v), and agar (2%, Dingguo Corp., Beijing, China; GA010-500 g) for 7 days at 30 °C. The conidia were harvested by washing PDA plates with distilled H_2_O containing 0.8% NaCl and 0.02% Tween 80. For preparation of protoplasts, 10^6^ conidia were inoculated in a 500-mL flask containing 200-mL liquid minimal medium (MM; [75]) and incubated at 30 °C overnight. The MM agar plate supplemented with 300 μg/mL hygromycin B and 1.5 mg/mL 5-FOA (Sigma, USA) was applied as the selective medium for the screening of the uracil auxotrophic transformants. Also, the MM agar plates containing Avicel (0.5%) or Avicel (0.5%) plus glucose (1.0%) as carbon resources were used to investigate the capacity of *T. reesei* strains to secrete cellulases. The CMC-esculin plate was used to screen the strains showing β-glucosidase (BGL) activity, and the medium composition was as follows: 3 g/L esculin, 10 g/L sodium carboxymethyl cellulose (CMC–Na), 0.5 g/L ferric citrate, and 20 g/L agar. To prepare the seed culture for cellulase production, the fungal spores (10^6^/mL) were inoculated in the liquid MM and incubated at 180 rpm and 30 °C for 36 h. Then, 10 mL of the inoculum was transferred to 100 mL of the cellulase production medium (CPM, [18]) in a 500-mL flask. The culture medium was supplemented with 0.1% uracil (Sigma, USA) when needed in the entire research process.

### Construction of the *cre1* deletion strain

Deletion of *cre1* with the *pyrG*+DR fragment in *T. reesei* was performed according to the method described previously [74]. To be specific, first, the *pyrG*+DR fragment containing the 2.8-kb *A. niger pyrG* gene and the 458-bp direct repeat (DR) sequence was constructed as follows: the *pyrG* gene was amplified by the primer pair pyrG-F/pyrG-R; then, the DR fragment was produced from the 3′ end of *pyrG* gene through PCR using the primer pair DR-F/DR-R and fused into the 5′ end of the *pyrG* gene through double-joint PCR [76]. The final *pyrG* + DR fragment was amplified by the nested primer pair, pyrG-UF1/pyrG-2425DR. Second, the upstream and downstream fragments of *cre1* were amplified using *T. reesei* genome as template by primer pairs cre1-UF/cre1-UR and cre1-DF/cre1-DR, respectively, and then fused together with the *pyrG*+DR fragment to construct the Δ*cre1::pyrG* cassette through Double-joint PCR. Finally, the primer pair cre1-F1/cre1-R1 was utilized as nested primers to amplify the entire Δ*cre1::pyrG* cassette (Fig. 1a).

The Δ*cre1::pyrG* cassette was transformed into SP4 protoplasts, and the fungal genomic DNA was isolated according to the methods described by Penttilä et al. [76]. The target transformants containing the *A. niger pyrG* gene were screened on MM containing 300 μg/mL hygromycin B, and the purified candidate transformants were identified through PCR by primer pairs, pyrG-UF1/pyrG-2425DR, cre1-2426UF/cre1-1059R, and cre1-497F/cre1-1059R. Afterward, 10^4^–10^6^ spores of three 5-FOA resistant transformants were spread onto MM plate containing 1 mg/mL uracil and 1.5 mg/mL 5-FOA to select the *pyrG* gene excision transformants by spontaneous mutation. Then, the purified candidate transformants were identified through PCR by primer pair, pyrG-UF1/pyrG-2425DR. Phanta^®^ Super-Fidelity DNA Polymerase (Vazyme Biotech Co., Ltd., Nanjing, China) was utilized for PCR amplification. The PCR primers were designed using the primer premier 5.0 software. The DNA fragments were recovered using Gel Extraction Kit (Omega, USA). Primers used for PCR analysis are listed in Table 1.

**Table 1:** Primers used in this study

Primers	Sequences (5′–3′)	Target gene
creF	GTACTTTGGCCCTCGCTGAG	*cre1*
creR	AGCAATCAGGTGCAGATATCAC	*cre1*
creRUTr	CCAGACTGCATAAGGATTCCC	*cre1*
cre1-2426UF	GCCAAGACTCAGCATAAA	*cre1*
cre1-1059R	GCTAATGATGTCGGTAAGT	*cre1*
cre1-497F	GCACTCCTACTCGTCCTT	*cre1*
cre1-UF	CCTTCAATGGGGAGGTGG	Upstream region of *cre1*
cre1-UR	TGGTGGGTGAGATAGACA	Upstream region of *cre1*
cre1-DF	CAGCACAATACGACTCCG	Downstream region of *cre1*
cre1-DR	TGCCGAATACCCTGAAAA	Downstream region of *cre1*
DR-F	TTGTCCCAGCCCGAGGCATTAG	DR fragment
DR-R	AGCCGCTGGTCAATGTTATC	DR fragment
cre1-F1	GGCGCCTGTGCCAGACTA	Δ*cre1::pyrG* cassette
cre1-R1	CAGCACAATACGACTCCG	Δ*cre1::pyrG* cassette
pyrG-F	CGCCGTCGTGTCTCGTCT	*A. niger pyrG*
pyrG-R	ACAGACGGGTATAGGGGTA	*A. niger pyrG*
cbh1-1138UF	CCTTTGGCGTTTCCCTGATTC	Promotor of *cbh1*
cbh1-F1	AAAGCGTTCCGTCGCAGTAG	Promotor of *cbh1*
cbh1-R1	AGCACGAGCTGTGGCCAAGAAGG	Promotor of *cbh1*
bglA-F1	CAATAGTCAACCGCGGACTGCGCATCATGAGGTTCACTTTGATCGAGGCG	*A. niger bglA*
bglA-R1	GTGGGTGGAGGGTGCTGGA	*A. niger bglA*
bg-F	GTATTACCCCTCCCCTTGG	*A. niger bglA*
bg-R	TCGTAGGCGATGTAGTGC	*A. niger bglA*
pyrG-UF1	AATGCTCCGTAACACCCA	*pyrG*+DR fragment
pyrG-2425DR	ATCATCGTAACCGAGAATCCA	*pyrG*+DR fragment
cre1-probe-F	GGACTTGACACGGGCTAT	Probe
cre1-probe-R	AGCCATCTCGCAGTGTAT	Probe

### Construction of the *A. niger bglA*-overexpressing strain

The *A. niger bglA* gene-overexpressing cassette was constructed through double-joint PCR. Chromosomal DNA of *A. niger* was used as the template to clone the β-glucosidase-encoding gene *bglA* (GenBank Accession No. AM270402) and its terminator region by primer pair bglA-F1/bglA-R1. Chromosomal DNA of *T. reesei* was used as the template to amplify the promotor of *cbh1* and its signal sequence by primer pair cbh1-F1/cbh1-R1. Then these two fragments were fused together through Double-joint PCR, and the final *A. niger bglA* gene-overexpressing cassette was amplified by primer pair cbh1-1138UF/bglA-R. The overexpressing cassette and the *pyrG*+DR fragment were co-transformed into the protoplasts of the Δ*cre1* strain SDC11 through the method mentioned above. The transformants were screened on MM containing 300 μg/mL hygromycin B, and the purified candidate transformants were identified through PCR by primer pairs cbh1-1138UF/bg-R and bg-F/bg-R.

### Enzyme activity and SDS-PAGE assay

Whatman no. 1 paper (Whatman, UK), CMC–Na (Sigma, USA), *p*-nitrophenyl-β-d-cellobioside (pNPC; Sigma, USA), and *p*-nitrophenyl-β-d-glucopyranoside (pNPG; Sigma, USA) were utilized to measure the filter paper (FP), endoglucanases (EGs), cellobiohydrolases (CBHs), and BGL activities, respectively, as described previously by Ghose [77]. One enzyme activity was defined as the amount of enzymes required to liberate one µmol glucose (FPA, EG) or *p*-nitrophenol (CBH, BGL) per minute under the assay conditions. The equal volume of culture supernatants were supplemented with loading buffer, boiled for 10 min for degeneration, and loaded onto a 12% SDS–polyacrylamide separating gel. Renaturing SDS-PAGE electrophoresis was performed in 12% SDS–polyacrylamide-separating gel using 0.3% CMC–Na as the substrate. Since it is difficult to separate the mycelial biomass from the insoluble cellulose substrate in the cellulase production medium, growth rates of *T. reesei* strains were measured by detecting the total intracellular protein amount. Specifically, the insoluble portion of the fungal culture was washed once with 0.7 NaCl and twice with distilled water and then extracted by 1 M NaOH. The NaOH extraction method and the correlation between the intracellular protein with the fungal growth were described previously [78, 79]. Analysis of protein concentration was performed using the Bio-Rad Protein Assay kit (Bio-Rad, USA).

### Southern blot analysis

The probe of *cre1* was a fragment amplified through PCR using the primer pair, cre1-probe-F/cre1-probe-R (Table 1) to detect the *cre1* gene. The *Eco*R I/*Hind* III-digested genomic DNA was separated in 0.8% agarose gel and transferred to a Hybond-N^+^ nylon membrane (Amersham, USA). DNA labeling and detection were performed using a DIG-High prime DNA labeling and detection starter kit (Roche, Germany) according to the manufacturer’s protocol.

### Transglycosylation reaction

The transglycosylation reaction was conducted according to the method described previously with some modifications [28]. Different concentrations of glucose (10, 20, 40, 60, and 80% (w/v)), dissolved in 0.05 M pH 4.8 citric acid buffer, were used as substrates in the transglycosylation reaction with the cellulase preparation from the *T. reesei* BGLA-overexpressing strain SCB18. The reaction was implemented at 65 °C in a 100-mL flask for 1d, 2d, and 3d in a total volume of 30 mL.

### TLC assay and glucose residual content detection

The silica gel 60 F254 plate (Millipore, Germany) was used for TCL assay [80]. In detail, all of the reaction solutions were diluted to 5% (w/v), and aliquots of the diluents (1 μL) were spotted 1.0 cm from the bottom of the plate. Then the plate was run using the developing agent containing butanol, isopropanol, acetic acid, and water, with their proportions in volume being fixed to 7:5:2:4, respectively. The plate was dried after completion of the assay, and the sugars were visualized after the plate was sprayed with the diphenylamine–aniline–phosphoric acid (DPA) reagent and reacted in the drying oven at 110 °C for 10 min. The glucose residual contents of the reaction solutions were further examined using the SBA-40C biological sensor analyzer (BISAS, Shandong, China).

### Cellulase production using the transglycosylation product

To determine the effect of the transglycosylation product on the induction of cellulase production, the liquid MM containing 2% glucose, 2% lactose, 2% Avicel, or 2% sugar-mixture (the transglycosylation product) was used as the carbon source to cultivate the carbon catabolite-derepressed strain *T. reesei* SDC11. The spores (10^6^/mL) were inoculated in a 500-mL flask containing 100 mL culture and incubated at 180 rpm, 30 °C for cellulase production.

### Saccharification of the pretreated corncob residues

Acid-pretreated and delignified corncob residues were applied as substrates for saccharification. The former residue contained 62.6% cellulose, 2.4% hemicellulose, 17.7% lignin, and 6.8% ash, while the latter one included 65.7% cellulose, 1.8% hemicellulose, 3.2% lignin, and 5.9% ash [43]. The saccharification reaction was implemented at 150 rpm and 50 °C in a 100-mL flask containing 5% (w/v) substrate. The fermentation broths with the equal FPA activity (10 FPU/g substrate) were loaded by adding pH 4.8 citric acid buffer to make up the total volume to 30 mL. The glucose release was detected using the SBA-40C biological sensor analyzer every 24 h. Cellulose conversion was calculated as follows:

$${\text{Cellulose conversion}}\;{ = }\;\frac{{{\text{Glucose}}\;{\text{yield}}\;{\text{from}}\;{\text{enzyme}}\;{\text{hydrolysis}}\; ( {\text{mg)}}}}{{{\text{Substrate}}\;{\text{weight}}\; ( {\text{mg)}} \times {\text{Cellulose}}\;{\text{content}}\; ({\text{\%)}}}} \times\,0. 9\,\times\,100 {\text{\%}}.$$

## Additional files

**Additional file 1: Figure S1.** *T. reesei* SP4 is carbon catabolite-repressed. **a** Growth of *T. reesei* SP4, QM9414 and RUT-C30 on the medium plate containing both glucose (1.0%) and Avicel (0.5%) as carbon sources. A clear cellulolytic halo was observed around the colony of RUT-C30, but not SP4 and QM9414. **b** Graphical representation of the *cre1* gene locus in the chromosomes of *T. reesei* QM9414 and RUT-C30. **c** PCR analysis of the internal fragment of the *cre1* gene in *T. reesei* SP4, QM9414 and RUT-C30 using the primer pair creF/creR. A 2.9-kb length fragment was amplified from QM9414 and SP4 but not from RUT-C30. **d** PCR analysis of the full-length *cre1* gene in *T. reesei* SP4, QM9414 and RUT-C30 using the primer pair creF/creRUTr. The primers provided a 1.9-kb fragment corresponding to the truncated *cre1* gene from RUT-C30, but yielded a larger fragment (4.4 kb) from QM9414 and SP4.

**Additional file 2: Figure S2.** PCR and phenotypic analysis of the Δ*cre1* strain *T. reesei* SDC11. **a** PCR analysis of *T. reesei* SDC11 with SP4 as control. 1 and 2 represent the fragment (upstream region and open reading frame of gene *cre1*) amplificated by the prime pair cre1-2426UF/cre1-1069R in *T. reesei* SDC11 and SP4, respectively; 3 and 4 represent the internal fragment of gene *cre1* amplificated by the prime pair cre1-497F/cre1-1069R in *T. reesei* SDC11 and SP4, respectively. 5 and 6 represent the fragment of gene *pyrG* amplificated by the prime pair pyrG-UF1/pyrG-2426DR in *T. reesei* SDC11 and SCP11, respectively. **b** Southern blot analysis of the genomic DNA isolated from SP4 and SCP11, which were digested with *Eco*RI/*Hin*dIII. A 5.5-kb fragment is present in the parental strain SP4, and a 7.0-kb band is shown in Δ*cre1* + *pyrG* strain SCP11. **c** Growth of *T. reesei* SN1, Δ*cre1* + *pyrG* strain SCP11 and Δ*cre1* strain SDC11 on MM plate. **d** Growth of *T. reesei* SP4, Δ*cre1* + *pyrG* strain SCP11 and Δ*cre1* strain SDC11 on the MM plate containing uracil (0.1%).

**Additional file 3: Figure S3.** Renaturing SDS-PAGE assay of BGL activities from the fermentation broths of *T. reesei* SDC11 and SP4 with equal FPA loading (0.04 FPA).

**Additional file 4: Figure S4.** PCR and phenotypic analysis of the *bglA* overexpression strain *T. reesei* SCB18. **a** Graphical representation of the *bglA* overexpression cassette. **b** PCR analysis of the *T. reesei* SCB18 and SDC11 strains. 1 and 3 represent the chimeric fragments spanning the *cbh1* promoter and the *bglA* gene, which were amplificated by the primer pair cbh1-1138UF/bg-R using the chromosomes of SCB18 and SDC11, respectively; 2 and 4 represent the internal fragments of the *bgl1 gene*, which were amplificated by the primer pair bg-F/bg-R using the chromosomes of SCB18 and SDC11, respectively. **c** Growth of *T. reesei* SP4, SDC11 and SCB18 on the medium plate containing Avicel (0.5%) as sole carbon source. **d** Growth of *T. reesei* SP4, SDC11 and SCB18 on the medium plate containing both glucose (1.0%) and Avicel (0.5%) as carbon sources.

**Additional file 5: Table S1.** Comparisons of BGL activity and cellulase titer in the *T. reesei* BGL-overexpressing strains.

## Abbreviations

BGLs: β-glucosidases
CBHs: cellobiohydrolases
EGs: endoglucanases
LPMOs: lytic polysaccharide monooxygenases
CCR: carbon catabolite repression
pNPG: 4-nitrophenyl-β-dglucopyranoside
pNPC: *p*-nitrophenyl-β-d-cellobioside
CMC–Na: sodium carboxymethyl cellulose
CPM: cellulase production medium
FPA: filter paper activity
SDS-PAGE: sodium dodecyl sulfate polyacrylamide gel electrophoresis
DPA: diphenylamine–aniline–phosphoric acid
TLC: thin-layer chromatography
