# Supplementary material for: Production of the versatile cellulase for cellulose bioconversion and cellulase inducer synthesis by genetic improvement of Trichoderma reesei
Source: Biotechnol Biofuels. 2017 Nov 15;10:272. doi: 10.1186/s13068-017-0963-1 (PMC5688634; doi:10.1186/s13068-017-0963-1)
Supplement: Supplementary file 1 — Additional file 1: Figure S1. T. reesei SP4 is carbon catabolite-repressed. a Growth of T. reesei SP4, QM9414 and RUT-C30 on the medium plate containing both glucose (1.0%) and Avicel (0.5%) as carbon sources. A clear cellulolytic halo was observed around the colony of RUT-C30, but not SP4 and QM9414. b Graphical representation of the cre1 gene locus in the chromosomes of T. reesei QM9414 and RUT-C30. c PCR analysis of the internal fragment of the cre1 gene in T. reesei SP4, QM9414 and RUT-C30 using the primer pair creF/creR. A 2.9-kb length fragment was amplified from QM9414 and SP4 but not from RUT-C30. d PCR analysis of the full-length cre1 gene in T. reesei SP4, QM9414 and RUT-C30 using the primer pair creF/creRUTr. The primers provided a 1.9-kb fragment corresponding to the truncated cre1 gene from RUT-C30, but yielded a larger fragment (4.4 kb) from QM9414 and SP4. [file 13068_2017_963_MOESM1_ESM.doc]

**Additional file 1**


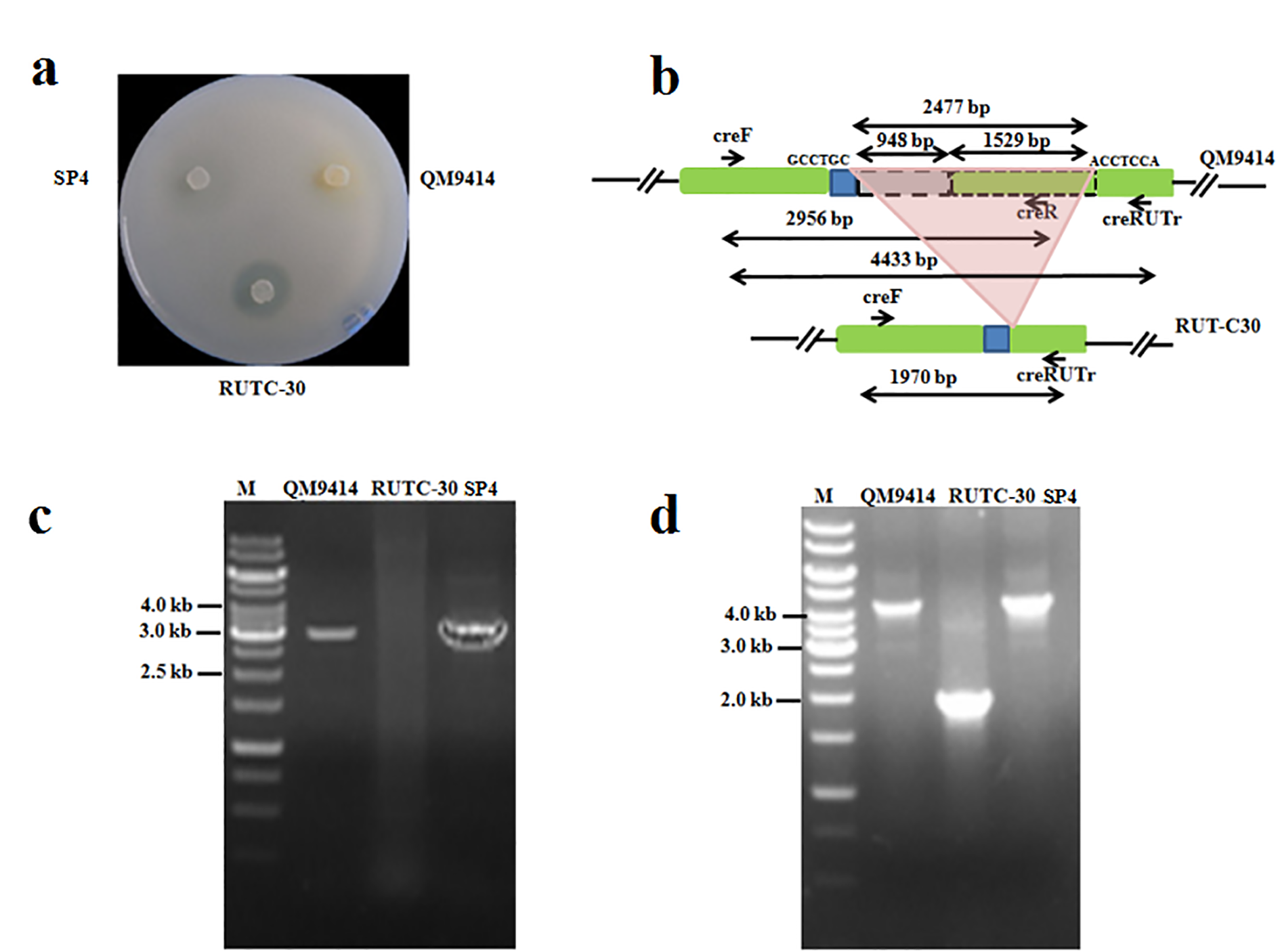


**Fig. S1** *T. reesei* SP4 is carbon catabolite-repressed. a Growth of *T. reesei* SP4, QM9414 and RUT-C30 on the medium plate containing both glucose (1.0%) and Avicel (0.5%) as carbon sources. A clear cellulolytic halo was observed around the colony of RUT-C30, but not SP4 and QM9414. b Graphical representation of the *cre1* gene locus in the chromosomes of *T. reesei* QM9414 and RUT-C30. c PCR analysis of the internal fragment of the *cre1* gene in *T. reesei* SP4, QM9414 and RUT-C30 using the primer pair creF/creR. A 2.9-kb length fragment was amplified from QM9414 and SP4 but not from RUT-C30. d PCR analysis of the full-length *cre1* gene in *T. reesei* SP4, QM9414 and RUT-C30 using the primer pair creF/creRUTr. The primers provided a 1.9-kb fragment corresponding to the truncated *cre1* gene from RUT-C30, but yielded a larger fragment (4.4 kb) from QM9414 and SP4.
