# Supplementary material for: Production of the versatile cellulase for cellulose bioconversion and cellulase inducer synthesis by genetic improvement of Trichoderma reesei
Source: Biotechnol Biofuels. 2017 Nov 15;10:272. doi: 10.1186/s13068-017-0963-1 (PMC5688634; doi:10.1186/s13068-017-0963-1)
Supplement: Supplementary file 2 — Additional file 2: Figure S2. PCR and phenotypic analysis of the Δcre1 strain T. reesei SDC11. a PCR analysis of T. reesei SDC11 with SP4 as control. 1 and 2 represent the fragment (upstream region and open reading frame of gene cre1) amplificated by the prime pair cre1-2426UF/cre1-1069R in T. reesei SDC11 and SP4, respectively; 3 and 4 represent the internal fragment of gene cre1 amplificated by the prime pair cre1-497F/cre1-1069R in T. reesei SDC11 and SP4, respectively. 5 and 6 represent the fragment of gene pyrG amplificated by the prime pair pyrG-UF1/pyrG-2426DR in T. reesei SDC11 and SCP11, respectively. b Southern blot analysis of the genomic DNA isolated from SP4 and SCP11, which were digested with EcoRI/HindIII. A 5.5-kb fragment is present in the parental strain SP4, and a 7.0-kb band is shown in Δcre1 + pyrG strain SCP11. c Growth of T. reesei SN1, Δcre1 + pyrG strain SCP11 and Δcre1 strain SDC11 on MM plate. d Growth of T. reesei SP4, Δcre1 + pyrG strain SCP11 and Δcre1 strain SDC11 on the MM plate containing uracil (0.1%). [file 13068_2017_963_MOESM2_ESM.doc]

**Additional file 2**

**
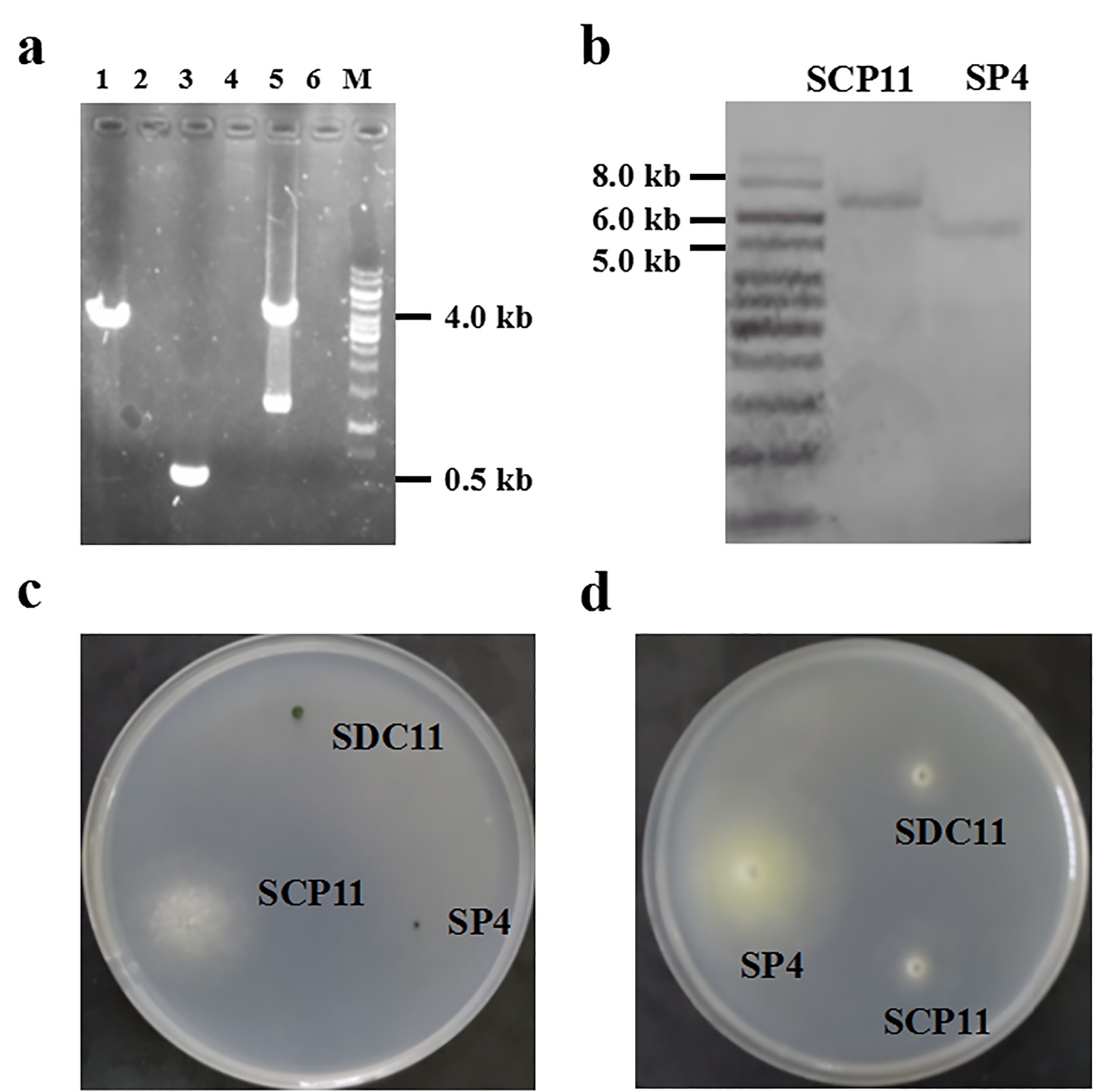
**

**Fig. S2** PCR and phenotypic analysis of the Δ*cre1* strain *T. reesei* SDC11**.** **a** PCR analysis of *T. reesei* SDC11 with SP4 as control. 1 and 2 represent the fragment ( upstream region and open reading frame of gene *cre1*) amplificated by the prime pair cre1-2426UF/cre1-1069R in *T. reesei* SDC11 and SP4, respectively; 3 and 4 represent the internal fragment of gene *cre1* amplificated by the prime pair cre1-497F/cre1-1069R in *T. reesei* SDC11 and SP4, respectively. 5 and 6 represent the fragment of gene *pyrG* amplificated by the prime pair pyrG-UF1/pyrG-2426DR in *T. reesei* SDC11 and SCP11, respectively. **b** Southern blot analysis of the genomic DNA isolated from SP4 and SCP11, which were digested with *Eco*R I/*Hin*d III. A 5.5-kb fragment is present in the parental strain SP4, and a 7.0-kb band is shown in Δ*cre1*+*pyrG* strain SCP11. **c** Growth of *T. reesei* SN1, Δ*cre1*+*pyrG* strain SCP11 and Δ*cre1* strain SDC11 on MM plate. **d** Growth of *T. reesei* SP4, Δ*cre1*+*pyrG* strain SCP11 and Δ*cre1* strain SDC11 on the MM plate containing uracil (0.1％).
