# Supplementary material for: Production of the versatile cellulase for cellulose bioconversion and cellulase inducer synthesis by genetic improvement of Trichoderma reesei
Source: Biotechnol Biofuels. 2017 Nov 15;10:272. doi: 10.1186/s13068-017-0963-1 (PMC5688634; doi:10.1186/s13068-017-0963-1)
Supplement: Supplementary file 3 — Additional file 3: Figure S3. Renaturing SDS-PAGE assay of BGL activities from the fermentation broths of T. reesei SDC11 and SP4 with equal FPA loading (0.04 FPA). [file 13068_2017_963_MOESM3_ESM.doc]

**Additional file 3**

**
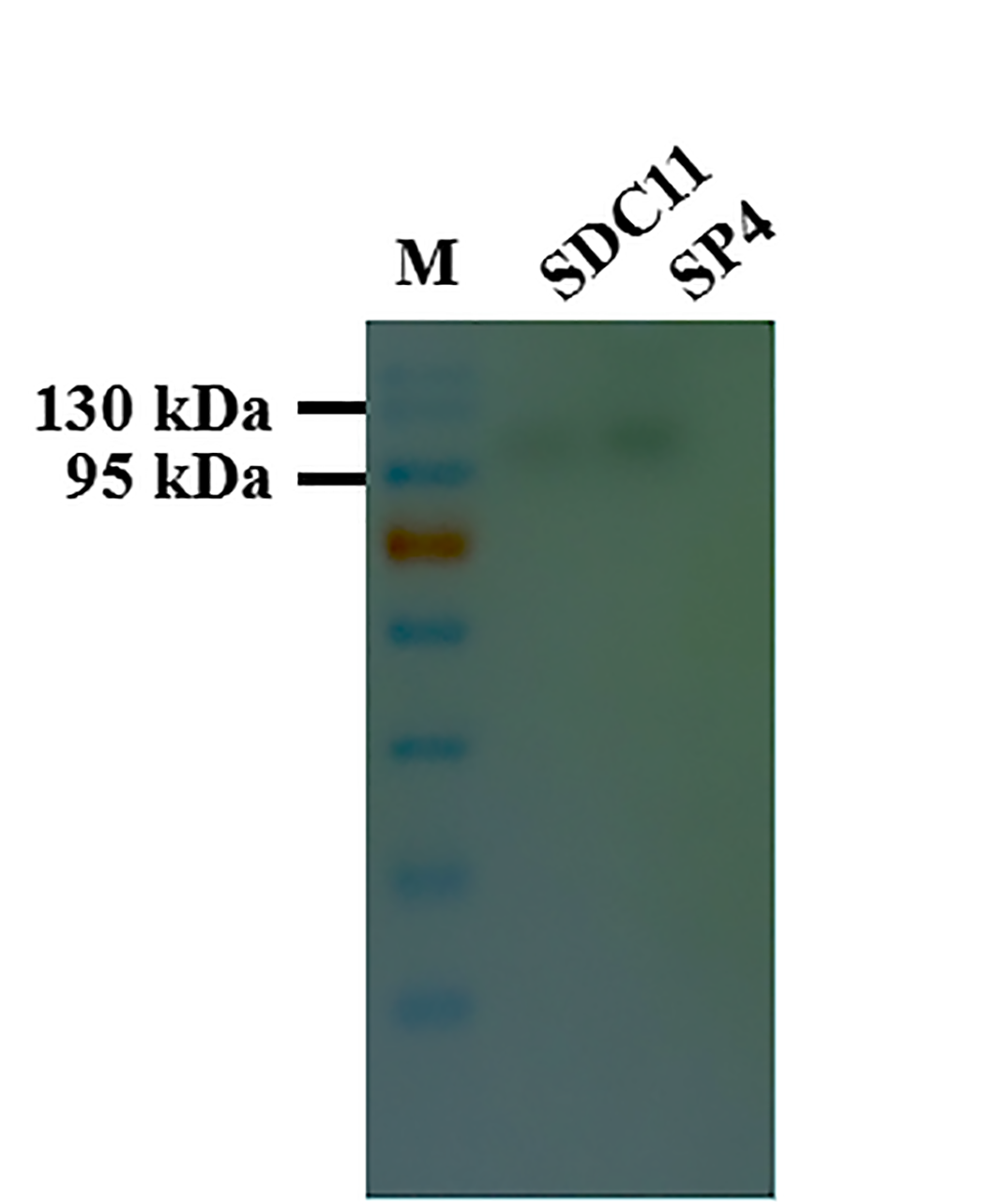
**

**Fig. S3** Renaturing SDS-PAGE assay of BGL activities from the fermentation broths of *T. reesei* SDC11 and SP4 with equal FPA loading (0.04 FPA).
