# Supplementary material for: Production of the versatile cellulase for cellulose bioconversion and cellulase inducer synthesis by genetic improvement of Trichoderma reesei
Source: Biotechnol Biofuels. 2017 Nov 15;10:272. doi: 10.1186/s13068-017-0963-1 (PMC5688634; doi:10.1186/s13068-017-0963-1)
Supplement: Supplementary file 4 — Additional file 4: Figure S4. PCR and phenotypic analysis of the bglA overexpression strain T. reesei SCB18. a Graphical representation of the bglA overexpression cassette. b PCR analysis of the T. reesei SCB18 and SDC11 strains. 1 and 3 represent the chimeric fragments spanning the cbh1 promoter and the bglA gene, which were amplificated by the primer pair cbh1-1138UF/bg-R using the chromosomes of SCB18 and SDC11, respectively; 2 and 4 represent the internal fragments of the bgl1 gene, which were amplificated by the primer pair bg-F/bg-R using the chromosomes of SCB18 and SDC11, respectively. c Growth of T. reesei SP4, SDC11 and SCB18 on the medium plate containing Avicel (0.5%) as sole carbon source. d Growth of T. reesei SP4, SDC11 and SCB18 on the medium plate containing both glucose (1.0%) and Avicel (0.5%) as carbon sources. [file 13068_2017_963_MOESM4_ESM.doc]

**Additional file 4**


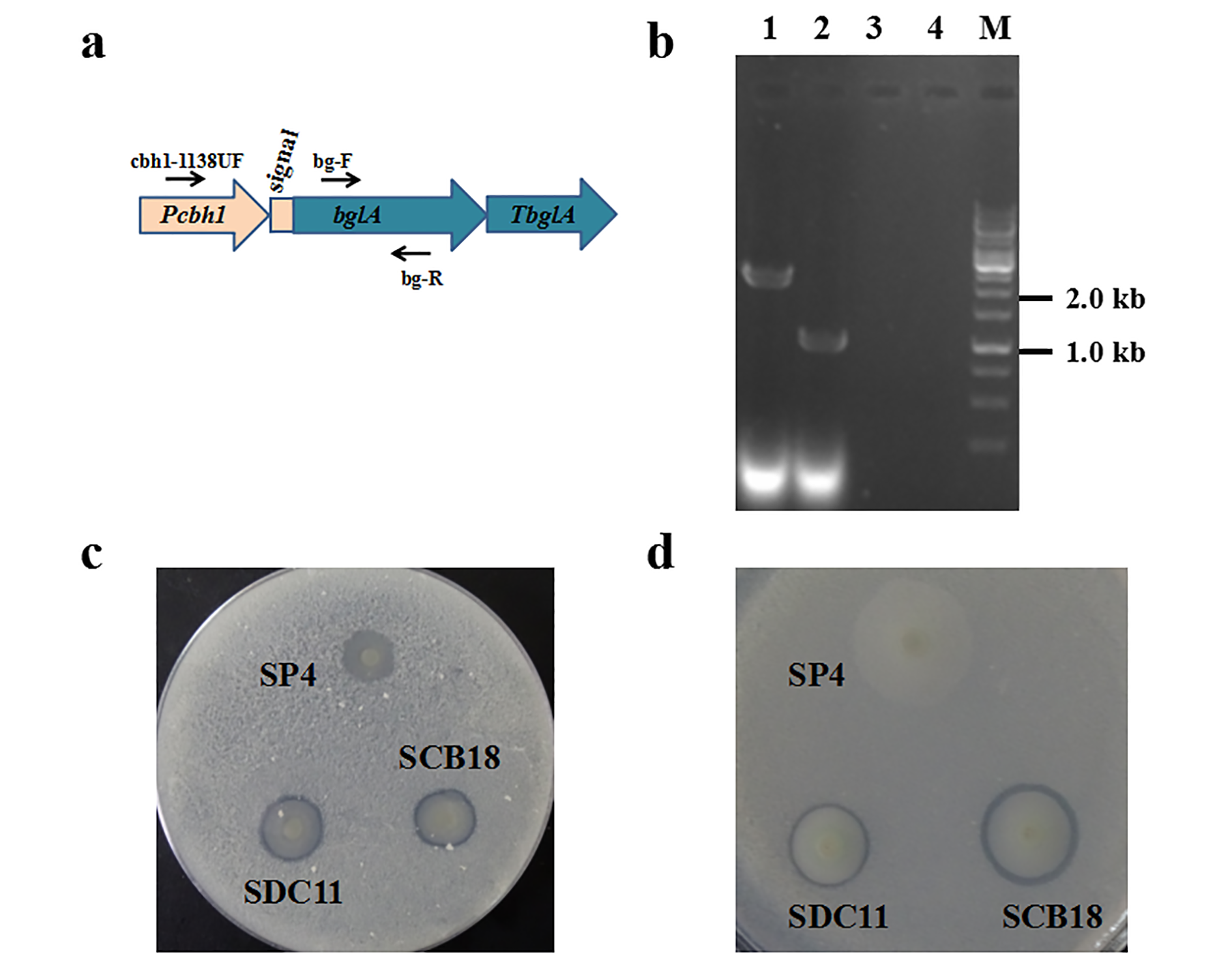


**Fig. S4** PCR and phenotypic analysis of the *bglA* overexpression strain *T. reesei* SCB18. **a** Graphical representation of the *bglA* overexpression cassette. **b** PCR analysis of the *T. reesei* SCB18 and SDC11 strains. 1 and 3 represent the chimeric fragments spanning the *cbh1* promoter and the *bglA* gene, which were amplificated by the primer pair cbh1-1138UF/bg-R using the chromosomes of SCB18 and SDC11, respectively; 2 and 4 represent the internal fragments of the *bgl1 gene*, which were amplificated by the primer pair bg-F/bg-R using the chromosomes of SCB18 and SDC11, respectively. **c** Growth of *T. reesei* SP4, SDC11 and SCB18 on the medium plate containing Avicel (0.5%) as sole carbon source. **d** Growth of *T. reesei* SP4, SDC11 and SCB18 on the medium plate containing both glucose (1.0%) and Avicel (0.5%) as carbon sources.
