# Supplementary material for: Production of the versatile cellulase for cellulose bioconversion and cellulase inducer synthesis by genetic improvement of Trichoderma reesei
Source: Biotechnol Biofuels. 2017 Nov 15;10:272. doi: 10.1186/s13068-017-0963-1 (PMC5688634; doi:10.1186/s13068-017-0963-1)
Supplement: Supplementary file 5 — Additional file 5: Table S1. Comparisons of BGL activity and cellulase titer in the T. reesei BGL-overexpressing strains. [file 13068_2017_963_MOESM5_ESM.doc]

Table S1

Comparisons of BGL activity and cellulase titer in the *T. reesei* BGL-overexpression strains.

| BGL donor | Host strain  (*T. reesei*) | Engineering strain  (*T. reesei*) | BGL activity  (IU/mL) | Cellulase titer  (FPA, IU/mL) | Carbon Source  (w/v) | Refs |
| --- | --- | --- | --- | --- | --- | --- |
| *A. niger* | SDC11 | SCB18 | 103.9 | 4.6 | 2% Avicel | This study |
| *T. reesei* | PC-3-7 | X3B1 | 0.90 | 2.1 | 2% Avicel | [23] |
| *P. decumbens* | RUT-C30 | P2# | 34.3 | 10.0 | 2% wheat bran, 3% microcrystalline cellulose | [20] |
| *A. niger* | ZU-02 | #6 | 5.3 | 4.33 | 2% lactose | [24] |
| *A. aculeatus* | PC-3-7 | JN11H | 10.0 | 1.7 | 1% Avicel | [60] |
| *T. reesei* | SN1 | SPB2 | 8.3 | 2.6 | 2% Avicel | [18] |
| *Neosartorya ficheri* | TU6 | T4 | 69.7 | 1.7 | 2% Avicel | [61] |
